# Clinical Characteristics and Outcomes of Patients With Diabetes Admitted for COVID-19 Treatment in Dubai: Single-Centre Cross-Sectional Study

**DOI:** 10.2196/22471

**Published:** 2020-12-07

**Authors:** Rahila Bhatti, Amar Hassan Khamis, Samara Khatib, Seemin Shiraz, Glenn Matfin

**Affiliations:** 1 Department of Endocrinology Mediclinic Parkview Hospital Dubai United Arab Emirates; 2 Department of Medicine Mohammed Bin Rashid University of Medicine and Health Sciences Dubai United Arab Emirates

**Keywords:** Diabetes, COVID-19, characteristic, outcome, chronic condition, cross-sectional

## Abstract

**Background:**

Recent studies have shown that diabetes is a major risk factor that contributes to the severity of COVID-19 and resulting mortality. Poor glycemic control is also associated with poor patient outcomes (eg, hospitalization and death).

**Objective:**

This study aimed to describe the clinical characteristics and outcomes of patients with diabetes who were admitted to our hospital for COVID-19 treatment.

**Methods:**

This cross-sectional, observational study comprised patients with diabetes admitted with COVID-19 to Mediclinic Parkview Hospital in Dubai, United Arab Emirates, from March 30 to June 7, 2020. We studied the differences among characteristics, length of hospital stay, diabetes status, comorbidities, treatments, and outcomes among these patients.

**Results:**

Of the cohort patients, 25.1% (103/410) had coexistent diabetes or prediabetes. These patients represented 17 different ethnicities, with 59.2% (61/103) from Asian countries and 35% (36/103) from Arab countries. Mean patient age was 54 (SD 12.5) years, and 66.9% (69/103) of patients were male. Moreover, 85.4% (88/103) of patients were known to have diabetes prior to admission, and 14.6% (15/103) were newly diagnosed with either diabetes or prediabetes at admission. Most cohort patients had type 2 diabetes or prediabetes, and only 2.9% (3/103) of all patients had type 1 diabetes. Furthermore, 44.6% (46/103) of patients demonstrated evidence suggesting good glycemic control during the 4-12 weeks prior to admission, as defined arbitrarily by admission hemoglobin A1_c_ level <7.5%, and 73.8% (76/103) of patients had other comorbidities, including hypertension, ischemic heart disease, and dyslipidemia. Laboratory data (mean and SD values) at admission for patients who needed ward-based care versus those who needed intensive care were as follows: fibrinogen, 462.8 (SD 125.1) mg/dL vs 660.0 (SD 187.6) mg/dL; D-dimer, 0.7 (SD 0.5) µg/mL vs 2.3 (SD 3.5) µg/mL; ferritin, 358.0 (SD 442.0) mg/dL vs 1762.4 (SD 2586.4) mg/dL; and C-reactive protein, 33.9 (SD 38.6) mg/L vs 137.0 (SD 111.7) mg/L. Laboratory data were all significantly higher for patients in the intensive care unit subcohort (*P*<.05). The average length of hospital stay was 14.55 days for all patients, with 28.2% (29/103) of patients requiring intensive care. In all, 4.9% (5/103) died during hospitalization—all of whom were in the intensive care unit.

**Conclusions:**

Majority of patients with diabetes or prediabetes and COVID-19 had other notable comorbidities. Only 4 patients tested negative for COVID-19 RT-PCR but showed pathognomonic changes of COVID-19 radiologically. Laboratory analyses revealed distinct abnormal patterns of biomarkers that were associated with a poor prognosis: fibrinogen, D-dimer, ferritin, and C-reactive protein levels were all significantly higher at admission in patients who subsequently needed intensive care than in those who needed ward-based care. More studies with larger sample sizes are needed to compare data of COVID-19 patients admitted with and without diabetes within the UAE region.

## Introduction

The burden of COVID-19, caused by the novel SARS-CoV-2, has being increasing worldwide. The World Health Organization declared COVID-19 a pandemic on March 11, 2020 [1]. As of October 6, 2020, a total of 99,733 COVID-19 cases have been reported in the United Arab Emirates with 429 COVID-19–related deaths [2].

Recent studies have suggested that diabetes is a major risk factor contributing to the severity of COVID-19 and resulting mortality [3]. For instance, a meta-analysis of 7 trials in China reported that 9.7% (153/1576) patients with COVID-19 had diabetes [4]. Diabetes is also considered a major risk factor for the development of severe pneumonia and clinical course resulting from COVID-19, and it is reported in approximately 20%-30% of such patients [5]. In addition, poor glycemic control, whether related to diabetes or stress hyperglycemia, is known to be associated with poor patient outcomes, including hospitalization and death [6]. A large population-based study in England reported 23,804 COVID-19–related deaths, of which one-third of the patients had diabetes (type 2 diabetes, n=7466, 31.4%; type 1 diabetes, n=365, 1.5%) [7].

Thus, the aim of this single-center cross-sectional study was to assess the clinical characteristics and outcomes of patients with diabetes admitted to a hospital in Dubai with moderate-to-severe COVID-19.

## Methods

### Study Design

This is a cross-sectional, observational study of patients with diabetes who were diagnosed with COVID-19 based on laboratory and/or radiological findings and admitted to Mediclinic Parkview Hospital, Dubai, United Arab Emirates, between March 30 and June 7, 2020.

### Definitions

A diagnosis of COVID-19 was confirmed by a positive COVID-19 reverse transcription–polymerase chain reaction (RT-PCR) test and/or consistent imaging findings from chest radiography or chest high-resolution computed tomography (HRCT), that is, radiological features of COVID-19 that are pathognomonic (eg, ground-glass opacity).

For the purposes of the study, the following definitions were considered:

- Diabetes was confirmed by either prior diagnosis or hemoglobin A1_c_ (HbA1_c_) ≥6.5% at admission.
- Prediabetes was confirmed by either prior diagnosis and/or HbA1_c_=5.7%–6.4% at admission.
- Uncontrolled diabetes was defined as HbA1_c_≥7.5% at admission.
- Patients were discharged from the hospital when they were clinically well and tested negative on two consecutive nasopharyngeal swab tests (laboratory tests) for COVID-19 RT-PCR.
- Admission laboratory tests were defined as tests performed within 24 hours of hospital admission.

### Data Collection

Data were collected from electronic medical records retrieved using the software Bayanaty (InterSystems IRIS). Information about patients’ basic demographics, nationalities, laboratory data, imaging findings (ie, chest radiograph and chest HRCT), and capillary blood glucose test performed at admission was extracted. Ethical approvals were received from the institutional research board of Mediclinic Parkview Hospital and Dubai Scientific Research Ethics Committee, Dubai Health Authority, Dubai, United Arab Emirates.

### Statistical Analysis

Statistical analyses were performed using SPSS software, version 25.0 (IBM Corp, released 2019). Frequencies with proportions were reported for categorical variables and mean values with standard deviation (SD) were reported for continuous variables. Mann Whitney test was used and a *P* value <.05 was considered statistically significant.

## Results

A total of 103 patients admitted to the hospital with a confirmed COVID-19 diagnosis had diabetes or prediabetes. During the same timeframe, overall, 410 patients with COVID-19 were admitted. Thus, 25.1% (103/410) of all patients admitted with COVID-19 had coexistent diabetes or prediabetes. Moreover, 66.9% (69/103) of the COVID-19 diabetes cohort were male patients. Patients within this study cohort represented 17 different ethnicities, of which 59.2% (61/103) were from Asian countries and 34.9% (36/103) were from Arab countries. The mean patient age was 54 (SD 12.5) years. In all, 73.8% (76/103) of the cohort patients had other comorbidities, including hypertension, ischemic heart disease, dyslipidemia, and chronic kidney disease (Table 1). Moreover, at admission, 9.7% (10/103) of the patients were receiving angiotensin converting enzyme inhibitor-I (ACE-I) treatment and 31.1% (32/103) patients were receiving angiotensin II receptor blocker (ARB) treatment, both of which were continued as per the individual patient’s clinical circumstances during admission, with no obvious adverse effects reported.

**Table 1:** Basic demographics and pre-comorbidities of study patients (n=103).

Study variable		Value	
**Gender, n (%)**			
	Male		69 (66.9)
	Female		34 (33.0)
Age in years, mean (SD)		54 (12.5)	
**Nationality, n (%)**			
	Arab		36 (34.9)
	Asian		61 (59.2)
	Western		5 (4.9)
	African		1 (0.9)
**Comorbidities^a^ (yes: n=76), n (%)**			
	Hypertension		66 (64.0)
	Dyslipidemia		54 (52.4)
	Ischemic heart disease		11 (10.6)
	Breast cancer		7 (6.8)
	Chronic kidney disease		4 (3.9)
	Stroke		3 (2.9)
	COPD^b^		1 (0.9)
	Renal transplant		1 (0.9)
**Medications, n (%)**			
	ACE-I^c^		10 (9.7)
	ARB^d^		32 (31.1)

^a^More than one comorbidity was reported for some patients.

^b^COPD: chronic obstructive pulmonary disease.

^c^ACE-I: angiotensin converting enzyme inhibitor.

^d^ARB: angiotensin II receptor blocker.

Out of the 103 cohort patients, 3 (2.9%) patients had type 1 diabetes and 90 (87.4%) patients had type 2 diabetes, 6 of whom were newly diagnosed with type 2 diabetes at admission. Moreover, 10 of 103 (9.7%) patients had prediabetes, 9 of whom were newly diagnosed at admission. Of those patients with a prior diagnosis of diabetes, 8 (7.8%) patients were on a basal insulin regimen and 6 (5.8%) patients were on basal-bolus insulin regimen (Table 2).

**Table 2:** Diabetes status among cohort patients before they acquired COVID-19 (n=103).

Item		Value, n (%)
**Type of diabetes or dysglycemic status**		
	Type 1	3 (2.9)
	Type 2	90 (87.4)
	Prediabetes	10 (9.7)
**Diabetes and dysglycemic status**		
	Known	88 (85.4)
	Unknown	15 (14.6)
**Control of diabetes**		
	Controlled (HbA1_c_^a^<7.5%)	46 (44.6)
	Not controlled (HbA1_c_≥7.5%)	52 (50.4)
**Diabetes medications taken prior to admission^b^**		
	Sulphonylurea	17 (16.5)
	Metformin	64 (62.1)
	Acarbose	1 (0.9)
	DDP-4^c^ inhibitor	4 (3.9)
	SGLT-2^d^ inhibitor	5 (4.9)
	GLP-1^e^ receptor agonist	1 (0.9)
	Basal insulin	8 (7.8)
	Basal-bolus insulin	6 (5.8)
Insulin dose (units) in 24 h, mean (SD)		73.4 (33.7)

^a^HbA1_c_: hemoglobin A1_c_.

^b^The same patient could have received more than one medication.

^c^DDP-4: dipeptidyl peptidase-4 inhibitor.

^d^SGLT-2: sodium-glucose co-transporter-2.

^e^GLP-1: glucagon-like peptide 1.

On admission, several laboratory investigations were performed (Table 3). Average random blood glucose was reported to be 10.2 mmol/L. Of the 103 patients; 56 (54.3%) had lymphopenia; 65 (63.1%) had received a confirmed diagnosis of pneumonia based on radiological findings; 38 (36.8%) had a normal chest radiograph, 14 of whom had confirmed pneumonia on chest HRCT; and 4 tested negative for COVID-19 based on laboratory RT-PCR but had radiologically confirmed COVID-19 pathognomonic changes.

**Table 3:** Laboratory data of study patients at hospital admission (n=103).

Laboratory test	Value at admission, mean (SD)	Normal range
HbA1_c_^a^ (%)	7.7 (1.6)	<5.6
Random blood glucose (mmol/L)	10.2 (4.2)	3.9-7.7
Hb (g/dL)	12.8 (1.7)	13.0-17.5
White blood cell count (10^3^/µL)	6.5 (3.1)	4.0-11.0
Lymphocyte count (10^3^/µL)	1.2 (0.7)	1.0-4.8
Lymphopenia, n (%)	56 (54.3)	N/A^b^
Platelets (10^3^/µL)	240.4 (85.3)	150-450
Creatinine (µmol/L)	92.7 (48.9)	63.6-110.5
GFR^c^ (mL/min/1.73^2^)	82.8 (22.2)	>60
Ferritin (ng/mL)	757.3 (1548.8)	21.8-274.7
Creatine kinase (U/L)	402.2 (1087.6)	30-200
LDH^d^ (U/L)	309.3 (228.9)	125-243
Fibrinogen (mg/dL)	529.3 (175.2)	200-400
Procalcitonin (ng/mL)	0.6 (1.2)	0.0-0.5
CRP^e^ (mg/L)	64.1 (82.7)	0.0-5.0
D-dimer (µg/mL)	1.1 (2.1)	0.0-0.5

^a^HbA1_c_: hemoglobin A1_c_.

^b^N/A: not applicable.

^c^GFR: glomerular filtration rate.

^d^LDH: lactate dehydrogenase.

^e^CRP: C-reactive protein.

Table 4 outlines emergent treatments used to manage patients with diabetes who were diagnosed with COVID-19 and their related outcomes. Of the 103 cohort patients, 50 (48.5%) patients received glucocorticoids at admission; 63 (61.2%) patients needed insulin at admission, requiring an average of 29.6 units per 24 h; and 5 (4.9%) patients had documented hypoglycemia (defined as blood glucose level <4 mmol/L) as detected by capillary blood glucose testing during admission (2 patients were on hydroxychloroquine, 4 on insulin, and 1 had end-stage renal disease).

**Table 4:** Emergent treatment and outcomes for cohort patients (n=103).

Item			Value
**Treatments, n (%)**			
	Glucocorticoids		50 (48.5)
	Hydroxychloroquine		82 (79.6)
	Antiviral medication: Kaletra (combination lopinavir/rapinavir)		54 (52.4)
	**Insulin administered on admission, n (%)**		63 (61.2)
		Intravenous insulin	10 (9.7)
		Subcutaneous insulin	53 (51.5)
	Units of insulin per 24 h, mean (SD)		29.6 (25.6)
**Outcomes**			
	**Hypoglycemia, n (%)**		5 (4.9)
		Insulin	4 (3.8)
		Hydroxychloroquine	2 (1.9)
		End-stage renal disease	1 (0.9)
	**Required ICU^a^, n (%)**		29 (28.2)
		Need ventilation, n (%)	12 (11.7)
		Need ECMO^b^, n (%)	2 (1.9)
		Total deaths (all in ICU), n (%)	5 (4.9)

^a^ICU: intensive care unit.

^b^ECMO: extracorporeal membrane oxygenation.

Of all the patients (29/103, 28.2%) who were admitted to the intensive care unit (ICU), 21 were from Asian countries and 8, from Arab countries. Furthermore, 22 patients were male. In all, 5 (4.9%) patients (2 males, 3 females) died—all were in the ICU. Four of these patients were of Asian origin, 3 were known to have type 2 diabetes, and 2 had received a new diagnosis of prediabetes on admission. Moreover, 2 of those 5 patients were obese, 1 had end-stage renal disease with known breast cancer, all 5 had pneumonia, and 4 developed acute respiratory distress syndrome with septic shock. Their average length of stay in the hospital was 18 days.

**Figure 1:**
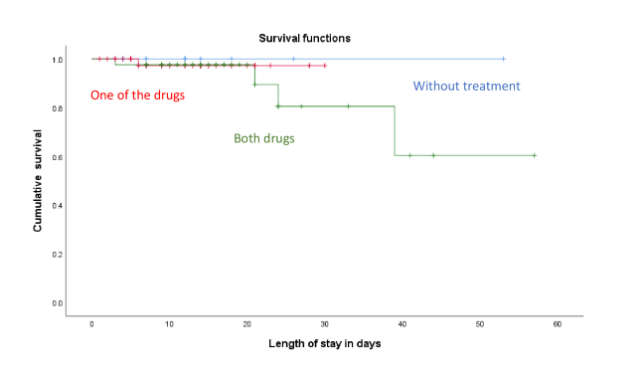
Survival curve generated using Kaplan-Meier method.

Kaplan-Meier survival curve (Figure 1) showed that 4 of the 5 deaths that occurred in our study cohort were of patients who received both hydroxychloroquine and glucocorticoid treatment (as might be anticipated based on the severity of disease). The median time of death was 21 (range 3-39) days.

### Subset Analysis

We performed a subanalysis of the patients’ laboratory data on admission and the maximum levels reported during the course of their hospital stay. Fibrinogen, D-dimer, and C-reactive protein (CRP) levels increased significantly during the hospital stay (Table 5).

Among the 103 patients who were admitted with diabetes or prediabetes and were diagnosed with COVID-19, 29 (28.1%) patients needed ICU care; these patients had significantly higher levels of fibrinogen, D-dimer, ferritin, and CRP at admission than did the patients managed by ward-based care (74/103, 71.8%; Table 6).

**Table 5:** Key biomarkers at admission and maximal levels during hospital stay (n=103).

Biomarkers	Values		
	At admission, mean (SD)	Maximum level during hospital stay, mean (SD)	*P* value
Fibrinogen (mg/dL)	529.3 (175.2)	550.1 (178.3)	<.001
Procalcitonin (ng/mL)	0.6 (1.2)	1.5 (3.2)	.12
CRP^a^ (mg/L)	64.1 (82.7)	92.2 (92.5)	<.001
D-dimer (µg/mL)	1.1 (2.1)	2.0 (3.9)	.01
Ferritin (ng/mL)	757.3 (1548.8)	521.0 (1898.9)	.06

^a^CRP: C-reactive protein

**Table 6:** Key admission biomarkers used as markers of COVID-19 severity (n=103).

Biomarkers	Values		
	Ward-based care (n=74), mean (SD)	ICU^a^ (n=29), mean (SD)	*P* value
Fibrinogen (mg/dL)	462.8 (125.1)	660.0 (187.6)	<.001
Procalcitonin (ng/mL)	0.8 (1.7)	0.6 (1.0)	.31
CRP^b^ (mg/L)	33.9 (38.6)	137.0 (111.7)	<.001
D-dimer (µg/mL)	0.7 (0.5)	2.3 (3.5)	.019
Ferritin (ng/mL)	358.0 (442.0)	1762.4 (2586.4)	.01

^a^ICU: intensive care unit

^b^CRP: C-reactive protein

## Discussion

In this single-center study conducted in Dubai, 25.1% (103/410) of patients with COVID-19 already had diabetes or prediabetes at admission. Of the study cohort, 14.6% (15/103) received a new diagnosis of diabetes or prediabetes at admission. According to the International Diabetes Federation, the prevalence of diabetes in the United Arab Emirates is 15.4% [8], and our data suggests an overrepresentation of diabetes and prediabetes as risk factors for more severe COVID-19 illness requiring admission.

Most patients with diabetes or prediabetes admitted to hospital for COVID-19 (69/103, 66.9%) were male. This finding is in line with emerging studies that show men with COVID-19 are at a higher risk for developing severe outcomes, including death, than women [3,4,9].

Dubai is a highly multinational society comprising people of more than 200 nationalities. The patients in our study cohort represented 17 different nationalities, of which 59.2% (61/103) were from Asian countries and 34.9% (36/103) were from Arab countries. This finding is also consistent with other studies that suggest that people of Black, Asian, and Minority Ethnic (BAME) populations have increased risk and are predisposed to worse clinical courses and outcomes with COVID-19 than are their Caucasian counterparts [10]. In addition, many individuals of BAME origin are also more likely to have diabetes or prediabetes [11].

Moreover, consistent with recent studies, most patients in our cohort with diabetes or prediabetes and COVID-19 (76/103, 73.8%) also had other notable comorbidities. This finding is consistent with the fact that diabetes and cardiovascular disease are key components of the metabolic syndrome [12]. The metabolic syndrome is also associated with proinflammatory and prothrombotic states, which may have important implications for COVID-19 cases, wherein such complications are especially common and troublesome [13].

In this study, laboratory test results showed that fibrinogen, D-dimer, ferritin, and C-reactive protein (CRP) levels at admission were higher in patients subsequently requiring intensive care treatment. This finding is reflective of the inflammatory cytokine response observed in COVID-19. In addition, measuring admission glucose levels and in-hospital monitoring are important. Acute hyperglycemia shows upregulation of *ACE2* gene, which facilitates entry of SARS-CoV-2 virus inside the cells. Prolonged hyperglycemia causes downregulation of *ACE2* expression, making the cells vulnerable to the inflammatory effects of the SARS-CoV-2 virus [14]. Thus, performing these laboratory tests at hospital admission may be useful for risk stratification, that is, to determine the potential severity of the disease and guide the level of care that may be required for the patient.

The limitations of our study include the small sample size and retrospective nature of our analysis. Therefore, more studies with larger sample sizes are needed. Another limitation of our study is that there was no control group of patients without diabetes for comparison of results (other than knowing the absolute numbers of total patients admitted with COVID-19 during the same timeframe). In addition, because of the increasing number of new patients presenting with COVID-19 symptoms to our healthcare facility over a short timeframe, data on accurate characterization of overweight or obese status were not available for the majority of our patients; these conditions are thus not reported as comorbidities despite excess bodyweight being related to more severe COVID-19 illness and increased mortality [3,4]. Laboratory investigations such as fibrinogen, D-dimer, ferritin, and CRP levels can help in the initial assessment of severity of disease and subsequent need for ward-based or intensive care.

### Conclusion

In this single-center study in Dubai, approximately 25% of patients admitted with COVID-19 also had diabetes or prediabetes. Most of these patients were male and of Asian origin, and 14.6% were newly diagnosed with diabetes or prediabetes upon admission. A majority of these patients (76/100, 73.8%) also had other notable comorbidities. Our comprehensive laboratory analyses revealed distinct abnormal patterns of biomarkers that are associated with a poor prognosis: fibrinogen, D-dimer, ferritin, and CRP levels were all significantly higher at admission in patients who subsequently needed intensive care than in those patients who required ward-based care. In all, 28.2% required ICU admission, of which 5 patients eventually died. More studies with larger sample sizes are needed to compare data of patients with COVID-19 admitted with and without diabetes in the UAE region.

## Abbreviations

ACE-I: angiotensin converting enzyme inhibitors
ACE2: angiotensin-converting enzyme 2
ARB: angiotensin II receptor blocker
BAME: Black, Asian, and Minority Ethnic
ECMO: extracorporeal membrane oxygenation
HBA1_c_: hemoglobin A1_c_
HRCT: high-resolution computed tomography
ICU: intensive care unit
RT-PCR: reverse transcription polymerase chain reaction
